# Involvement of Oxidative and Endoplasmic Reticulum Stress in *RDH12*-Related Retinopathies

**DOI:** 10.3390/ijms22168863

**Published:** 2021-08-18

**Authors:** Hajrah Sarkar, Maria Toms, Mariya Moosajee

**Affiliations:** 1 Institute of Ophthalmology, University College London, London EC1V 9EL, UK; h.sarkar@ucl.ac.uk (H.S.); maria.toms.14@ucl.ac.uk (M.T.); 2 The Francis Crick Institute, London NW1 1AT, UK; 3 Moorfields Eye Hospital NHS Foundation Trust, London EC1V 2PD, UK; 4 Great Ormond Street Hospital for Children NHS Foundation Trust, London WC1N 3JH, UK

**Keywords:** retinol dehydrogenase 12, oxidative stress, endoplasmic reticulum stress, autophagy, zebrafish, all-trans retinal, pregabalin

## Abstract

Retinol dehydrogenase 12 (RDH12) is expressed in photoreceptor inner segments and catalyses the reduction of all-trans retinal (atRAL) to all-trans retinol (atROL), as part of the visual cycle. Mutations in *RDH12* are primarily associated with autosomal recessive Leber congenital amaurosis. To further our understanding of the disease mechanisms, HEK-293 cell lines expressing wildtype (WT) and mutant *RDH12* were created. The WT cells afforded protection from atRAL-induced toxicity and oxidative stress. Mutant *RDH12* cells displayed reduced protein expression and activity, with an inability to protect cells from atRAL toxicity, inducing oxidative and endoplasmic reticulum (ER) stress, with upregulation of *sXBP1*, *CHOP*, and *ATF4*. Pregabalin, a retinal scavenger, attenuated atRAL-induced ER stress in the mutant *RDH12* cell lines. A zebrafish *rdh12* mutant model (*rdh12^u533^* c.17_23del; p.(Val6AlafsTer5)) was generated through CRISPR-Cas9 gene editing. Mutant fish showed disrupted phagocytosis through transmission electron microscopy, with increased phagosome size at 12 months post-fertilisation. Rhodopsin mislocalisation and reduced expression of *atg12* and *sod2* indicated early signs of a rod-predominant degeneration. A lack of functional RDH12 results in ER and oxidative stress representing key pathways to be targeted for potential therapeutics.

## 1. Introduction

Leber congenital amaurosis (LCA) is a severe early onset autosomal recessive retinal dystrophy, and mutations in the retinol dehydrogenase 12 (*RDH12)* gene (OMIM: 608830) account for approximately 10% of all cases [1]. *RDH12* is located on chromosome 14, consisting of seven exons and encoding a protein that is 35 kDa, with the majority of mutations being missense. *RDH12*-LCA is a progressive condition that typically presents with night blindness, nystagmus, and central loss of vision in early childhood, leading to complete blindness in adulthood [2]. Autosomal dominant mutations in *RDH12* have also been linked to a later onset milder retinitis pigmentosa (RP) phenotype [3,4].

*RDH12* is expressed in photoreceptor inner segments and is responsible for the reduction of all-trans retinal (atRAL) to all-trans retinol (atROL), as part of the visual cycle [5]. atRAL is primarily reduced to atROL in photoreceptor outer segments (POSs) by RDH8; however, in periods of intense illumination, atRAL leaks to the inner segments where it is reduced by RDH12 [6]. RDH12 can also act on medium chain aldehydes, suggesting a possible role in the detoxification of lipid peroxidation products [7]. In COS-7 cells transfected with various *RDH12* missense variants, mutant protein was expressed at lower levels and displayed significantly reduced enzyme activity, compared with wildtype (WT) RDH12 [8].

Two *Rdh12* knockout mouse models were generated; however, both displayed a relatively mild phenotype, with normal retinal histology and no apparent signs of retinal degeneration. Maeda et al. [9] did, however, report an accumulation of atRAL and slower electroretinogram (ERG) recovery after bleaching with increased susceptibility to light induced damage. Although there was no accumulation of atRAL in a second *Rdh12^-/-^* mouse model, retinal homogenates displayed decreased capacity to reduce atRAL, and increased levels of the retinal pigment epithelium (RPE) lipofuscin bis-retinoid *N*-retinyl-*N*-retinylidene ethanolamine (A2E) in *Rdh12^-/-^* eyes at 3–6 months [10,11]. Knockout *Rdh12* mouse models do not recapitulate the severe phenotype observed in patients; therefore, the study of disease mechanisms remains a challenge. It is thought that a lack of functional RDH12 results in a build-up of atRAL in the photoreceptors, causing toxicity and resulting in retinal degeneration.

atRAL is a highly reactive molecule and, if not reduced, is toxic to cells inducing oxidative stress [12,13]. In *Abca4^-/-^Rdh8^-/-^* mice, light-induced damage resulted in increased atRAL, which led to overproduction of reactive oxygen species (ROS), which was prevented by pre-treatment with Ret-NH2, a retinoid cycle inhibitor and retinal scavenger [14]. Treatment of ARPE-19 cells with atRAL resulted in a significant increase in intracellular ROS and upregulation of *NRF2*, *HO-1*, and *γ-GCSh*. Nrf2 (nuclear factor E2-related factor 2) is a transcription factor that regulates the cells’ response to oxidative stress through the expression of antioxidant and detoxifying genes. Under normal conditions, Nrf2 expression in the cytoplasm is controlled by Keap1. Cysteine residues on Keap1 detect ROS levels in the cells, and under high ROS levels, a confirmational change in Keap1 releases Nrf2, which translocates to the nucleus and binds to the antioxidant response element in the promoter region of antioxidant and phase II detoxifying enzymes, like heme oxygenase 1 (HO-1), NAD(P)H Quinone Dehydrogenase 1 (NQO-1), glutathione peroxidase (GPX), superoxide dismutase (SOD), and catalase (CAT) [15,16].

atRAL has also been shown to induce ER stress [13,17], which leads to an accumulation of misfolded proteins in the ER, triggering the unfolded protein response pathway (UPR). The UPR is activated through three sensors: PKR-like ER kinase (PERK), inositol requiring enzyme 1 (IRE1), and activating transcription factor 6 (ATF6), which are associated with binding immunoglobulin protein (BiP/GRP78). Upon activation of these sensors by autophosphorylation, they dissociate from BiP, triggering a cascade of events resulting in inhibition of protein translation, chaperone expression, and protein degradation [18]. Activation of IRE1 leads to splicing of X-box protein 1 (*XBP1*), resulting in *sXBPI*, which activates UPR genes responsible for protein folding, trafficking, and degradation. Activation of PERK results in activation of ATF4, leading to upregulation of UPR genes. Activation of ATF6 results in its translocation to the Golgi, where it is cleaved and then translocated back to the nucleus, where it induces expression of target genes, including chaperones. The role of these pathways is to alleviate ER stress and restore ER homeostasis; however, when ER stress becomes overwhelmingly high, ATF4 activates C/EBP homologous protein (CHOP), which in turn triggers apoptosis [19,20].

There are currently no treatments available for *RDH12*-related retinopathies, and little is known about the disease mechanisms. In this paper, we have created two disease models: (i) HEK-293 cell lines expressing WT and mutant *RDH12* and (ii) an *rdh12* mutant CRISPR-Cas9 zebrafish model. We have identified a number of pathways that appear to be disrupted, including oxidative stress, ER stress, and autophagy, representing potential future therapeutic targets.

## 2. Results

### 2.1. Generation of HEK-293 Stable Cell Lines Expressing Wildtype and Mutant RDH12 

**Figure 1:**
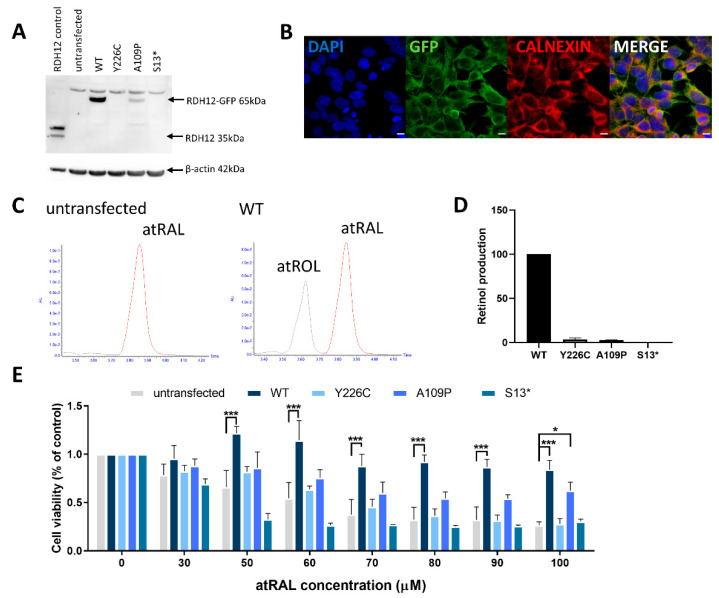
Generation of HEK-293 stable cell line expressing wildtype and mutant RDH12. (**A**) Western blot analysis of HEK-293 cells transfected with GFP-tagged RDH12. p.Y226C and p.S13* cell lines show no RDH12 protein expression, while p.A109P shows reduced expression. (**B**) Co-staining of calnexin (red) and GFP (green) confirmed localisation of RDH12 to the endoplasmic reticulum. Nuclei were counterstained with DAPI (4′,6-diamidino-2-phenylindole) (blue). Scale bar = 10 µM. (**C**) RDH12 activity assay using HPLC showed no atROL was detected in untransfected cells, but it was found in WT cells, confirming active functional RDH12. (**D**) Enzyme activity of all mutant proteins was significantly reduced compared with WT RDH12. (**E**) RDH12 protects against atRAL-induced toxicity. Cells were dosed with increasing concentrations of atRAL for 24 h and cell viability was assessed by MTT assay. atRAL is toxic to untransfected cells, whereas cells expressing WT RDH12 were protected from atRAL-induced cell death. p.Y226C and p.S13* mutant RDH12 did not protect cells from atRAL toxicity, whereas p.A109P mutant protein offered significantly higher protection than untransfected cells at 100 µM atRAL concentration. Three independent experiments were performed. Data are expressed as mean ± SEM, and analysed using two-way ANOVA, followed by Dunnetts multiple comparison test. * *p* ≤ 0.05, *** *p* ≤ 0.001.

As there are currently no available cell lines natively expressing RDH12, we created a HEK-293 cell line expressing WT RDH12. HEK-293 cells were transfected with a vector encoding *RDH12* with a C-terminal green fluorescent protein (GFP) tag. Western blot was used to evaluate the expression of RDH12 (Figure 1A), with a band at approximately 65 kDa corresponding to RDH12-GFP. Calnexin staining confirmed the native localisation of RDH12 to the ER (Figure 1B). High-performance liquid chromatography (HPLC) was used to determine the activity of RDH12. WT RDH12 protein exposed to atRAL resulted in the production of atROL, compared with protein from untransfected cells, where no atROL was detected (Figure 1C). This confirmed that the stable cell line expresses WT RDH12, which is correctly localised to the ER, and is active.

In order to study the disease mechanisms and screen potential therapeutics for *RDH12*-retinopathies, three mutant *RDH12* stable HEK-293 cell lines were created; two expressing missense mutations (p.Y226C and p.A109P) and one nonsense mutation (p.S13*). Site-directed mutagenesis was used to introduce mutations into the WT *RDH12* plasmid, and mutagenesis was confirmed by Sanger sequencing. Western blot probing for RDH12 was used to analyse levels of protein expression. The p.Y226C mutant showed no protein expression, consistent with previous publications [21]. Cells expressing the p.A109P mutant showed reduced expression of RDH12, whereas the p.S13* mutant showed no protein expression, consistent with it being a null mutant, thus no full length protein is produced (Figure 1A). The HPLC activity assay showed significantly reduced RDH12 activity in the p.Y226C (3.5 ± 1.6%) and p.A109P lines (2.7 ± 0.3%) (*p* < 0.0001) and no activity in the p.S13* line (Figure 1D).

### 2.2. RDH12 Protects Cells from All-Trans Retinal-Induced Toxicity

High concentrations of atRAL are toxic to cells. Hence, in order to determine the ability of RDH12 to protect cells against this stressor, and whether mutant cells retain this ability, increasing concentrations of atRAL were added to cells for 24 h and cell viability was assessed by MTT assay. In untransfected cells, increasing concentrations of atRAL resulted in a dose-dependent decrease in cell viability. However, in cells expressing WT RDH12, no significant decrease in cell viability was observed with atRAL up to 100 µM (Figure 1E). At 100 µM atRAL, WT cells offered significant protection with 84% cell viability compared to untransfected cells, which displayed only 26% viability (*p* < 0.001). In all mutant lines, decreased cell viability was observed compared to WT at concentrations of 50 µM atRAL and above, consistent with the reduced expression and activity of mutant RDH12. In cells expressing the p.Y226C and p.S13* mutants, no increased protection against atRAL toxicity was observed compared to the untransfected cells. However, the p.A109P mutant cells did offer increased protection (64% cell viability) against 100 µM atRAL toxicity compared to the untransfected cells (*p* = 0.023), consistent with reduced expression levels.

### 2.3. atRAL Induces Oxidative Stress in Mutant Cell Lines

**Figure 2:**
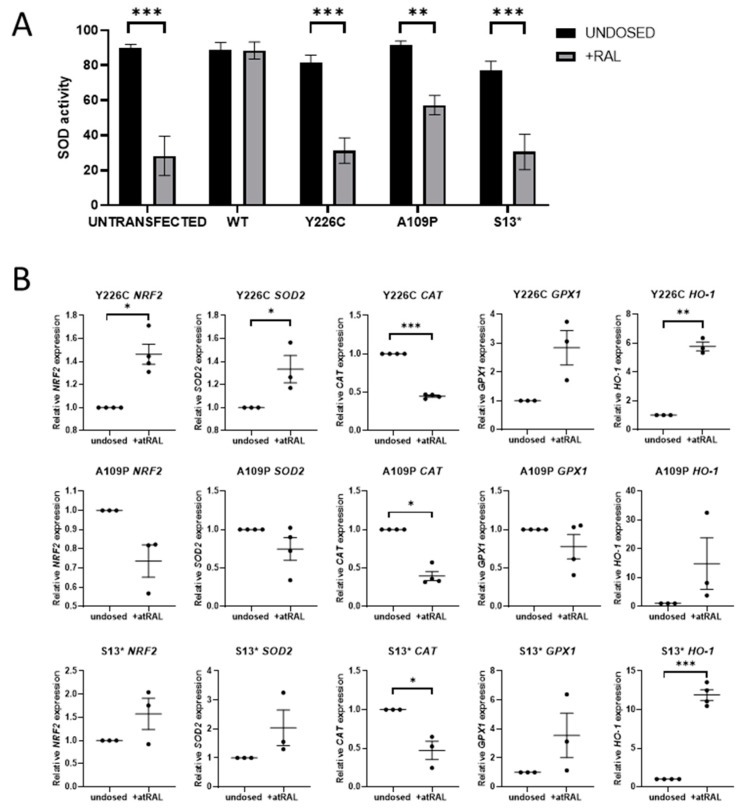
RDH12 protects cells from atRAL-induced oxidative stress. (**A**) Dosing untransfected cells with 50 µM atRAL for 24 h causes a significant reduction in SOD activity, indicating an increase in oxidative stress. Cell expressing WT RDH12 are protected from atRAL-induced oxidative stress. Dosing with atRAL causes a significant reduction in SOD activity in mutant cells. Statistical significance was analysed using two-way ANOVA and Sidak’s multiple comparison test. ** *p* ≤ 0.01, *** *p* ≤ 0.001. (**B**) Expression of oxidative stress markers *NRF2, SOD2, CAT, GPX1,* and *HO-1* was analysed by RT-qPCR following treatment with 50 µM atRAL for 24 h. Statistical significance was analysed with paired *t*-test. * *p* ≤ 0.05, ** *p* ≤ 0.01, *** *p* ≤ 0.001.

Excess atRAL has been shown to induce oxidative stress in ARPE-19 cells, resulting in an increase in ROS [13]. SOD is the first line of defence antioxidant enzyme that dismutates superoxide anions to hydrogen peroxide. SOD activity was measured following dosing with atRAL for 24 h. In untransfected cells, this significantly reduced SOD activity (*p* < 0.0001), indicating an increase in ROS beyond the capacity of SOD enzymes, resulting in oxidative stress. In contrast, SOD activity was not affected by addition of atRAL in WT RDH12 cells, demonstrating that protection was conferred by the WT protein. SOD activity was reduced in all three mutant lines, indicating an increase in oxidative stress levels and impairment of the compensatory defence mechanisms (Figure 2A). mRNA expression of oxidative stress markers was also analysed by RT-qPCR in mutant cell lines treated with atRAL, which resulted in a significant increase in *NRF2* in the p.Y226C line (*p* = 0.0132) and similarly in *HO-1* in the p.Y226C and p.S13* mutant lines (*p* = 0.004 and *p* = 0.0006, respectively). *CAT* mRNA expression was significantly reduced in all three mutant cell lines (Figure 2B). N-acetylcysteine amide (NACA), an antioxidant, was tested for its ability to reduce atRAL-induced oxidative stress. NACA was used at a concentration of 750 µM based on previous publications [22,23]. Treatment with 750 µM NACA for 24 h did not restore atRAL induced reduction in SOD activity, nor did it significantly attenuate atRAL-induced changes in *NRF2* and *CAT* expression. However, NACA did show a general trend with reducing atRAL-induced upregulation of *HO-1* expression in all mutant lines and significantly in the p.S13* line (*p* = 0.0173) (Supplementary Materials Figure S1).

### 2.4. Pregablain Attenuates atRAL-Induced ER Stress in RDH12 Mutant Cell Lines

**Figure 3:**
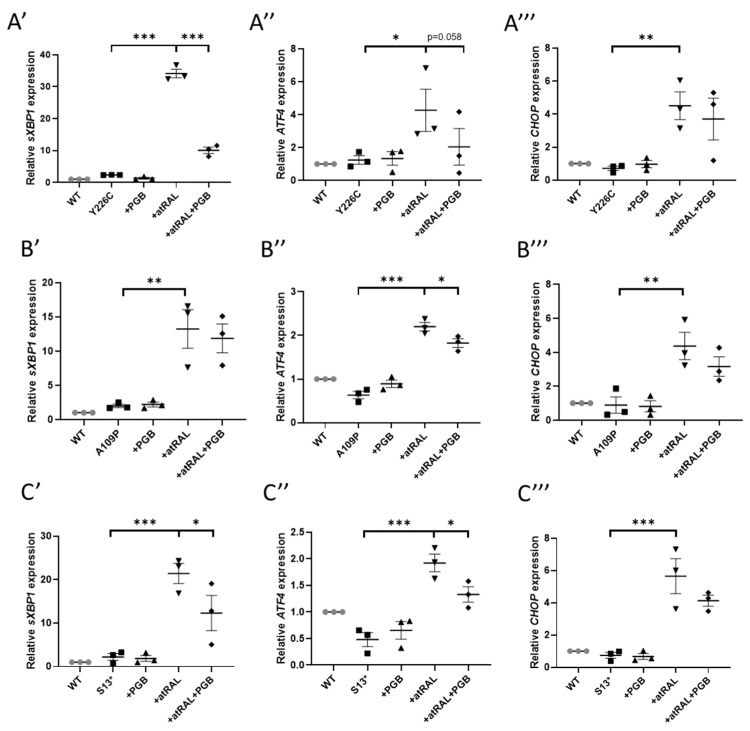
atRAL-induced ER stress was attenuated by pregabalin. Cells were dosed with 50 µM atRAL, 1 mM pregabalin (PGB), or both for 24 h. RT-qPCR was performed analysing the expres-sion of ER stress markers. Dosing with atRAL significantly increased expression of sXBP1 (A′, B′, C′), ATF4 (A″, B″, C″), and CHOP (A‴, B‴, C‴) in p.Y226C (**A**), p.A109P (**B**), and p.S13 (**C**) cell lines. Dosing with pregabalin reduced expression of ER stress markers. Statistical significance was analysed using one-way ANOVA and Sidak’s multiple comparison test. * *p* ≤ 0.05, ** *p* ≤ 0.01, *** *p* ≤ 0.001.

Expression of *sXBPI* through RT-qPCR was slightly increased in all mutant lines compared to WT RDH12 cells, with a 2.3-, 2-, and 2.2-fold increase in p.Y226C, p.A109P, and p.S13* cells, respectively. This could be a result of RDH12 misfolding caused by the mutations, triggering the UPR pathway. Incubation with 50 µM atRAL for 24 h resulted in a significant increase in expression of *sXBP1* by 14.6-, 6.6-, and 9.6-fold in the p.Y226C, p.A109P, and p.S13* cells, respectively, compared to undosed cells. Treatment with atRAL also significantly increased expression of *CHOP* by 6.3, 5.2 and 7.6-fold and *ATF4* by 3.4-, 3.5-, and 4-fold in p.Y226C, p.A109P, and p.S13* lines, respectively (Figure 3).

Pregabalin is a primary amine containing drug, commonly used to treat epilepsy, nerve pain, and anxiety [24], and was shown to protect *Rdh12^-/-^* knockout mice from light-induced retinal degeneration [25]. As shown in Figure 3, co-treatment of 1 mM pregabalin with atRAL for 24 h significantly reduced expression of *sXBP1* in the p.Y226C (*p* < 0.0001) and p.S13* (*p* = 0.0178) lines, and reduced expression of *ATF4* in all mutant cell lines. These data show that pregabalin can reduce ER stress. NACA also significantly reduced the expression of *sXBP1*, *CHOP*, and *ATF4* in p.S13* line (Supplementary Materials Figure S2). Tauroursodeoxycholic acid (TUDCA), a drug that has ER stress inhibiting properties, was also tested. Cells were treated with TUDCA at various concentrations (25–300 µM) for 24 h and cell viability was assessed to determine the optimal dose. A concentration of 100 µM TUDCA was chosen, as it was well tolerated by the cells (95% cell viability), with a minimal final concentration of 0.5% dimethyl sulfoxide (DMSO). TUDCA did not reduce atRAL-induced ER stress in any of the mutant cell lines (Supplementary Materials Figure S3).

### 2.5. Generation and Characterisation of rdh12^u533^ Zebrafish

**Figure 4:**
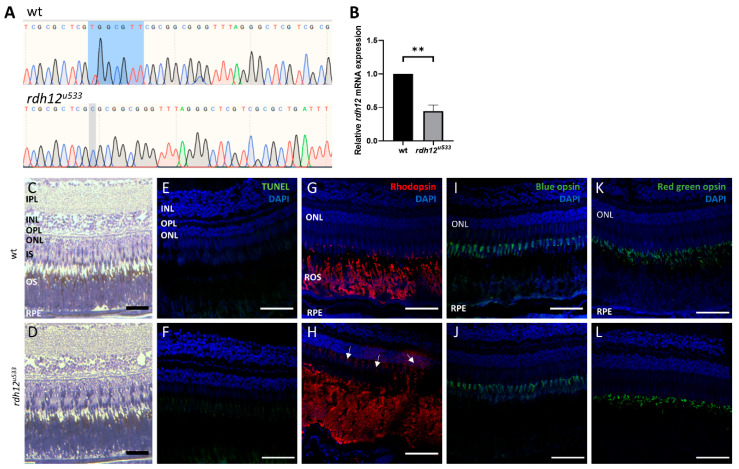
Generation and characterisation of *rdh12^u533^* mutant zebrafish. (**A**) Sanger sequencing traces showed a 7 bp deletion (c.17_23del) in the *rdh12^u533^* mutant fish. The deleted bases are highlighted in blue on the wildtype trace. (**B**) RT-qPCR showed a significant reduction of *rdh12* mRNA expression in the *rdh12^u533^* fish at 5 dpf (** *p* ≤ 0.001 analysed by paired *t*-test). (**C**,**D**) Retinal sections from 12 mpf fish were stained with toluidine blue to assess retinal structure. (**E**,**F**) TUNEL assay revealed no cell death Immunohistochemistry staining was used to detect rhodopsin (red) (**G**,**H**), blue opsin (green) (**I**,**J**), and red/green opsin (green) (**K**,**L**). Sections were counterstained with DAPI (blue). Rhodopsin mislocalisation was observed in *rdh12^u533^* fish. Scale bar = 50 µM. IPL, inner plexiform layer; INL, inner nuclear layer; OPL, outer plexiform layer; ONL, outer nuclear layer; IS, inner segment; OS, outer segment; RPE, retinal pigment epithelium.

**Figure 5:**
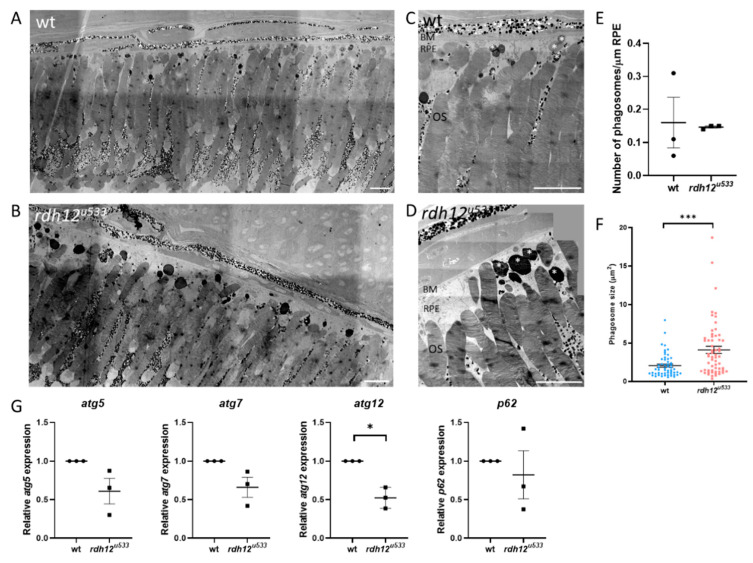
Retinal ultrastructure of wt and *rdh12^u533^* mutant fish. Transmission electron microscopy was used to assess retinal ultrastructure of wt (**A**,**C**) and *rdh12^u533^* (**B**,**D**) fish at 12 mpf. Phagosomes are indicated with white asterisks. Scale bar = 50 µm. (**E**) No significant difference was noted in the number of phagosomes between wt and *rdh12^u533^* fish. (**F**) Phagosomes were significantly larger in the *rdh12^u533^* fish at 12 mpf. Data are displayed as mean ± SEM. Phagosome number and size was quantified using ImageJ from three wt and *rdh12^u533^* fish. Statistical significance was analysed by Mann–Whitney, *** *p* ≤ 0.001. (**G**) mRNA expression of autophagy genes in the retina of 12 mpf fish was analysed by RT-qPCR. Expression of *atg12* was significantly reduced in the *rdh12^u533^* fish. * *p* ≤ 0.05. OS, outer segment; RPE, retinal pigment epithelium; BM, Bruch’s membrane.

**Figure 6:**
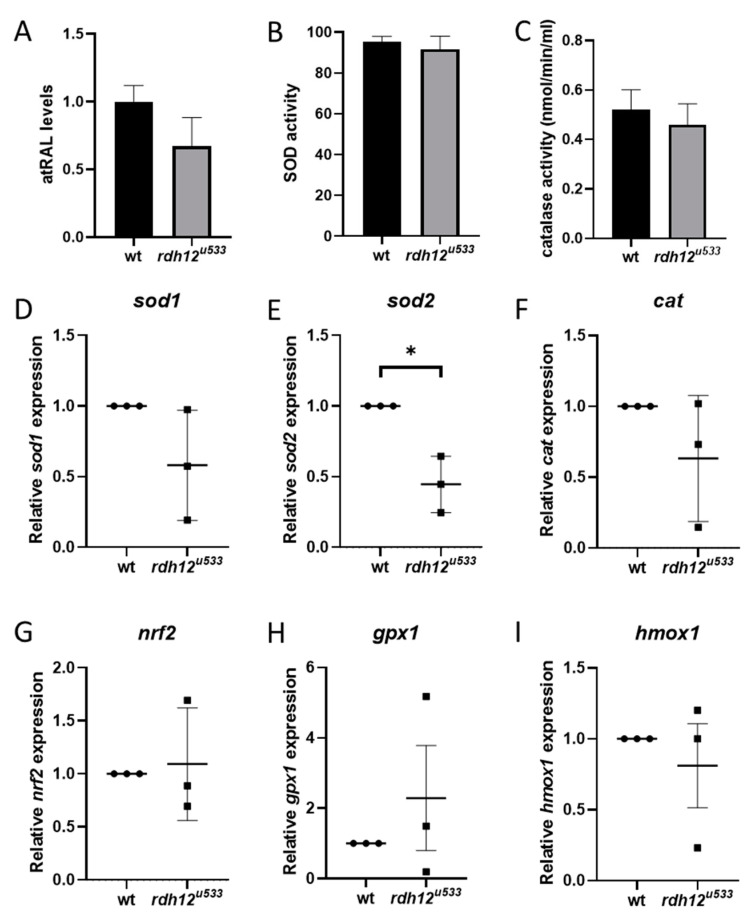
Analysis of oxidative stress in adult *rdh12^u533^* zebrafish retina. (**A**) atRAL levels in 16 mpf retina were analysed by HPLC. Activity of antioxidant enzymes SOD (**B**) and CAT (**C**) was analysed in 16 mpf retina, but no significant differences were found between wt and *rdh12^u533^* mutant fish. (**D**–**I**) Expression of oxidative stress markers was analysed by RT-qPCR in retinas from wt and *rdh12^u533^* fish at 12 mpf. mRNA expression of *sod2* was significantly reduced in *rdh12^u533^* fish retinas. Data are expressed as mean ± SEM; * *p* < 0.05.

An in vivo *rdh12* zebrafish mutant model was generated using CRISPR-Cas9 mutagenesis. The *rdh12^u533^* mutant line carried a 7 base pair (bp) deletion c.17_23del; p.(Val6AlafsTer5), which leads to a premature termination codon in exon 1 (Figure 4A). Expression of *rdh12* mRNA transcript was significantly reduced in the mutant fish at 5 days post-fertilisation (5 dpf) (*p* = 0.0087) (Figure 4B). Embryos were characterised through retinal histology, TUNEL assay, and rhodopsin immunostaining, but no evidence of a disease phenotype was observed up to 5 dpf, with no difference in SOD activity or atRAL levels between wildtype (wt) and *rdh12^u533^* fish (Supplementary Materials Figure S4). At 12 months post-fertilisation (12 mpf), retinal histology and TUNEL assay in the *rdh12^u533^* fish were akin to wt controls. However, rhodopsin was mislocalised to the photoreceptor inner segments and outer nuclear layer in the *rdh12^u533^* retina (Figure 4H). Blue and red/green opsin expression was similar between the wt and *rdh12^u533^* fish (Figure 4I–L). Retinal ultrastructure was examined using transmission electron microscopy (TEM), which revealed a significant increase in the size of phagosomes in the mutant RPE (*p* = 0.0001) (Figure 5F). Expression of autophagy markers was reduced in the *rdh12^u533^* fish, with *atg12* significantly reduced by 1.9-fold compared with the wt fish (*p* = 0.0038) (Figure 5G). No significant difference in atRAL levels was observed in the 16 mpf retinas between the wt and *rdh12^u533^* fish (Figure 6A). There was no significant difference in SOD and CAT activity at 16 mpf, although mRNA expression of *sod2* was significantly reduced (*p* = 0.0085) at 12 mpf in the *rdh12^u533^* fish (Figure 6E). No significant difference in expression of ER stress markers at 12 mpf was observed between the wt and *rdh12^u533^* fish (Supplementary Materials Figure S5).

## 3. Discussion

Mutations in *RDH12* are associated with severe early onset retinal degeneration. However, owing to the lack of phenotype observed in the *Rdh12^-/-^* mouse models, the exact disease mechanism of *RDH12*-retinopathies is not known. Lack of functional RDH12 is thought to result in a build-up of atRAL, which is toxic to cells and induces a number of apoptotic pathways. In this study, HEK-293 cell lines expressing WT and mutant RDH12 were created to study the effects of RDH12 dysfunction. WT RDH12 protected cells against atRAL-induced cell death and oxidative stress, and mutant RDH12 lost this protective capability, resulting in a decrease in cell viability and an increase in atRAL-induced oxidative and ER stress. We generated a CRISPR-Cas9 *rdh12* mutant zebrafish model that had a late-onset disease phenotype showing evidence of mild/early rod photoreceptor degeneration with associated RPE changes.

Several studies have linked a build-up of atRAL to an accumulation of ROS in ARPE-19 cells, leading to oxidative stress [12,13,14,17]. Exposure of *Abca4^-/-^ Rdh8^-/-^* mice to bright light induced atRAL build-up, which also led to an increase in ROS in the photoreceptors [14]. Exposure of ARPE-19 cells to atRAL resulted in upregulation of *NRF2* and antioxidant genes *HO-1* and *y-GCSh* [13]. Similarly, atRAL treatment increased the expression of *NRF2* in the p.Y226C cell line, and increased *HO-1* expression in all mutant lines. The redox state of the cell is controlled by a fine balance between the level of ROS and antioxidants. Oxidative stress is a result of an imbalance between ROS and antioxidant levels. Under mild stress, antioxidant enzymes scavenge ROS to maintain homeostasis in the cell. However, if ROS levels become too high, cellular homeostasis is disrupted and antioxidant enzymes become overwhelmed as they cannot cope with the high ROS load [26]. The three main classes of antioxidant enzymes are SOD, CAT, and GPX. A significant reduction in *CAT* mRNA expression and SOD enzyme activity was observed in atRAL-treated mutant cells, indicating that the antioxidant defence mechanisms of the cell are impaired and cannot protect the cells against the high free radical load. In the *rdh12^u533^* zebrafish retina at 12 mpf, *sod2* expression was significantly reduced by 2.2-fold compared with wt fish. Selective knockout of *Sod2* in the RPE of mice resulted in reduced ERG c-wave peak and increased RPE cell area in the central retina. EM also revealed disorganised cristae and enlarged mitochondria in the RPE. Increased oxidative stress was seen in the photoreceptors and increased *Nrf2* expression in the RPE/choroid complex of *Sod2* mice [27]. In another *Sod2* RPE knockdown mouse model, suppression of *Sod2* also resulted in increased oxidative stress, loss of ERG response, shortened inner and outer segments, and a thinner outer nuclear layer [28]. Overall, SOD activity was not affected in the *rdh12^u533^* fish up to 16 mpf, and expression of other antioxidant genes (*sod1, cat, gpx1,* and *hmox-1)* did not differ significantly between wt and *rdh12^u533^* retinas, suggesting that sod1 and other antioxidant enzymes can compensate for the reduced *sod2* expression at this timepoint, thereby preventing oxidative stress and cellular damage. Possible later timepoints may reveal further disturbances as the disease progresses.

ROS have been shown to co-localise with mitochondria and the ER in atRAL-treated ARPE-19 cells and induce ER stress via activation of the PERK-eIF2α-ATF4 pathway with increased expression of ER stress markers *BiP, ATF4*, and *CHOP* [13]. Upregulation of CHOP via activation of ATF4 triggers apoptosis [29]. Similarly, in our study, incubation with atRAL resulted in an increase in mRNA expression of *sXBP1, ATF4*, and *CHOP* in all mutant lines, indicating that atRAL activated ER stress induced apoptosis. Pregabalin lowered expression of ER stress markers in mutant HEK-293 cells. ER stress and differences in atRAL levels were not seen in the *rdh12^u533^* fish at 12 mpf, so pregabalin was not tested in the zebrafish model. Pregabalin is a primary amine containing drug, which can bind free atRAL via a Schiff base and lower its concentration in the retina, thereby preventing the formation of A2E. Pregabalin reduced atRAL levels in *Rdh12^-/-^* mice [25], and reduced ROS production in light-exposed *Abca4^-/-^ Rdh8^-/-^* mice [14]. Pregabalin also had a neuroprotective effect in a rat model of diabetic retinopathy, by reducing retinal glutamate, nitric oxide, and malondialdehyde and increasing glutathione levels, and restored retinal histology, by increasing retinal thickness and ganglion cell layer count [30]. Pregabalin is commonly used to treat epilepsy, nerve pain, and anxiety [24]. The most common adverse effects reported with pregabalin use are dizziness, sleeplessness, blurred vision, impaired concentration, dry mouth, oedema, and weight gain [24]. However, recently, the Medicines and Healthcare Products Regulatory Agency (MHRC) have reported severe respiratory depression linked to pregabalin [31].

In the *rdh12^u533^* zebrafish retina at 12 mpf, we found mislocalised rhodopsin, larger phagosomes, and reduced expression of autophagy markers with a significant decrease in *atg12* mRNA expression. Atg5 was found to co-localise with phagosomes containing POS in control mouse RPE. RPE-specific deletion of *Atg5* in mice resulted in a reduced ERG response at 16 weeks of age. *Atg5*-RPE knockout mice also showed accumulation of phagosomes with undigested POS and the POS were not able to penetrate the RPE beyond the apical surface. These mice also had a higher level of 11-cis, all-trans, and 13-cis retinal [32]. In a zebrafish model of *USH2A*-related RP, the *ush2a^rmc1^* mutant, disruption in rhodopsin localisation and autophagy was also noted, with increased autophagosomes and increased gene expression of *atg5* and *atg12* and LC3 protein expression [33]. Similar to *RDH12*-related RP, the retinal degeneration in *USH2A* patients is later onset, developing from adolescence, compared to those with LCA. Kim et al. [32] suggested that phagocytosis of the POS by the RPE and the visual cycle converge via a non-canonical autophagy pathway, both of which are thought to be disrupted in *RDH12*-related retinopathies. In this pathway, termed LC3-associated phagocytosis (LAP), following phagocytosis of the POS, the phagosome associates with the Atg12-Atg5-Atg16L complex, triggering lipidation of microtubule-associated protein 1 light chain 3 (LC3) to LC3-II and its recruitment to the phagosome. This signals for the lysosome to fuse with the phagosome, forming a phagolysosome, resulting in degradation of ingested POS and its recycling back to the photoreceptors. This pathway converges with the visual cycle, as POS phagocytosis aids in recycling of the visual pigments back to the photoreceptors. TEM imaging revealed enlarged phagosomes in the RPE of *rdh12^u533^* fish, indicating a disruption of RPE phagocytosis, with inefficient digestion of POS. Disruption of phagocytosis along with a build-up of retinoids leads to the formation of lysosomal bodies called lipofuscin. The retinoid pigments in lipofuscin are autofluorescent, and are seen on fundus imaging [34]. *RDH12*-LCA patients have widespread RPE atrophy and fundus imaging typically shows central hypoautofluorescence corresponding to RPE cell death, surrounded by hyperautofluorescent lesions, indicating regions of high toxicity and diseased RPE [35].

In conclusion, through the generation of two models of *RDH12*-disease, an in vitro human cell line and an in vivo zebrafish mutant model, we have seen that mutations in *RDH12* result in an inability of the enzyme to reduce atRAL, leading to toxic levels of this retinoid in cells. This triggers apoptosis, through induction of the oxidative stress and ER stress pathways. Although no gross abnormalities were noted in retinal histology in the *rdh12^u533^* fish and atRAL levels were comparable to wt fish, early indicators of disrupted pathways are observed in the adult fish including rhodopsin mislocalisation and increased phagosomal size with changes in autophagy related expression, suggesting that stress in the retina is effectively controlled so as not to lead to apoptosis or severe disruption at least up till 12–16 months of age. This corresponds more with the autosomal dominant *RDH12*-associated RP phenotype, which is a later-onset and relatively mild phenotype in comparison to the severe early-onset LCA phenotype. A similar difference between the human and zebrafish phenotypes has been observed previously in the modelling of *KCNJ13*-LCA, where the homozygous zebrafish *kcnj13^td15^* showed an adult-onset retinal degeneration, contrasting with the early severe phenotype seen in patients [36]. It is possible that compensatory mechanisms exist in the zebrafish, accounting for these differences. Although no other *rdh12* isoforms exist in the zebrafish, several retinol dehydrogenases are present in the retina that may compensate for lack of rdh12 in zebrafish. In addition, zebrafish are a cone-dominant species, and the cone-specific visual cycle, which is independent of the RPE, may play a more prominent role in the regeneration of the visual chromophore in zebrafish. As the mouse models of *RDH12* disease also do not show a severe phenotype, a species specific role may exist for RDH12 in humans that is either redundant or compensated for in other species. Further monitoring of the fish at later time points will provide more insight into the *rdh12* phenotype. We have identified a number of key pathways to be targeted for potential therapeutics, including pregabalin. Drugs with ER stress lowering properties, in addition to antioxidants and retinal scavengers, represent a new class of potential drugs that can be targeted for *RDH12*-related retinopathies.

## 4. Materials and Methods

### 4.1. Stable HEK-293 Cell Line Generation

The ORF expression clone was obtained from GeneCopoeia (pEZ_M98), which encoded *RDH12* (NM_152443) with a C-terminal GFP tag. Three known patient mutations (c.677A > G, p.Y226C; c.325G > C, p.A109P; and c.38C > A, p.S13*) were introduced into plasmids using the Quickchange II Site-Directed Mutagenesis kit (Agilent, Santa Clara, CA, USA). Primers were designed, according to kit instructions, with approximately 10 bases of correct sequence either side of the mutation (Supplementary Materials Table S1). Colonies with the correct mutation were verified by Sanger sequencing.

HEK-293 cells were cultured in DMEM high glucose, 10% FBS, and penicillin/streptomycin (Thermo Fisher Scientific, Waltham, MA, USA), and medium was changed every 3–4 days. Cells were passaged at 80–90% confluency using TrypLE Express (Thermo Fisher Scientific).

Cells were plated in six-well plates at a density of 600,000 cells per well and, 24 h later, they were transfected using Lipofectamine 2000 Reagent (Thermo Fisher Scientific). Twenty-four hours after transfection, cells were passaged into 96-well plates in selection media (DMEM high glucose, 10% FBS, 1 mg/mL G418) using serial dilutions to achieve low cell density. After 10 days, all non-transfected cells died off and, after a further 2 weeks, transfected cells formed distinct colonies. Individual colonies were picked into 24-well plates and expanded. This was defined as passage 0. For continued culture, G418 concentration was reduced to 0.5 mg/mL. All further experiments were carried out on cells from passage 5 onwards.

### 4.2. Western Blot

Cells were lysed in RIPA buffer (Thermo Fisher Scientific)(city, state abbreviation if USA or Canada, country)), and the protein concentration was quantified using BCA Protein Assay kit (Thermo Fisher Scientific). Samples were analysed by Western blot as previously described [37], using anti-RDH12 (1:1000) overnight at 4 °C. Anti-RDH12 and RDH12 transfected HEK293T cell lysate for use as a positive control were kindly gifted by Professor Debra Thompson (University of Michigan). Membrane was stripped and re-probed with anti-β-actin (A2228; 1:5000; Sigma-Aldrich, St. Louis, MO, USA), as a loading control.

### 4.3. Immunocytochemistry

Cells were fixed in chamber slides with 4% PFA/PBS for 20 min at 4 °C; permeabilised in 100% cold methanol for 5 min at room temperature (RT); and blocked for 1 h in 10% normal goat serum (NGS), 0.1% X-100 in PBS at RT. Cells were incubated overnight at 4 °C with primary antibodies diluted in 1% NGS at RT. Alexa Fluor (Thermo Fisher Scientific) secondary antibodies (1:400) were added for 1 h at RT. Slides were washed and mounted with Prolong Diamond with DAPI and imaged on LSM 710 confocal microscope (Leica Microsystems, Vienna, Austria). The primary antibodies used were anti-Calnexin (610524;1:100; BD Biosciences, Wokingham, UK) and anti-GFP (ab290;1:200; Abcam, Cambridge, UK).

### 4.4. RDH12 Activity Assay

Cell pellets were resuspended in 1× PBS, lysed by sonication, and centrifuged at 13,000 rpm for 10 min. Supernatant was collected and the protein concentration was determined using BCA Protein Assay kit. Reactions were carried out in 500 µL PBS, using 50 µg protein, 1 mM NADPH (Cayman Chemical, Ann Arbor, MI, USA), and 2.5 mM atRAL (Sigma R2500). Reaction was initiated with the addition of NADPH and incubated at 37 °C for 15 min, and stopped by the addition of 500 µL cold methanol. Retinoids were extracted according to the protocol by Chetyrkin et al. [38] on Waters Sep-Pak C18 column. HPLC analysis was performed using a Waters ACQUITY^®^ UPLC^®^ (Waters, Wilmslow, UK). Elution was monitored at 325 nm for atROL and 360 nm for atRAL with a Waters ACQUITY 2996 PDA. The stationary phase was Waters ACQUITY UPLC BEH C18 1.7 μm, 2.1 × 50 mm column and the mobile phase consisted of acetonitrile/water 0.1% formic acid (87.5:12.5). The analysis was 8 min, with a flow rate of 0.25 mL/min and a 10 μL injection.

### 4.5. Cell Viability MTT Assay

Cells were plated at a density of 40,000 cells per well in a 96-well plate. Cells were incubated with various concentrations of atRAL for 24 h. Cell viability was determined using MTT (M6494; Thermo Fisher Scientific). Following dosing, treatment media was replaced with 100 µL fresh media. MTT was prepared at a concentration of 5 mg/mL in PBS, and 10 µL was added to each well. Cells were incubated for 4 h at 37 °C. All but 25 µL media was removed, and MTT was solubilised with 50 µL DMSO. Absorbance was read at 540 nm on a Safire2 plate reader (TECAN, Männdorf, Switzerland).

### 4.6. Drug Dosing

For all dosing experiments, atRAL was used at a concentration of 50 µM. Pregabalin (Sigma) was used at a concentration of 1 mM, NACA (Tocris Bioscience, Bristol, UK) at 750 µM, and TUDCA (Cayman Chemical) at 100 µM. Cells were plated in six-well plates at a density of 700,000 cells per well. After 24 h, drugs were added to the cells in culture media. Twenty-four hours later, cells were pelleted and stored at −80 °C for further analysis.

### 4.7. RT-qPCR

Total RNA was extracted from cells using the RNeasy mini kit and from whole embryos and enucleated adult eyes using the RNeasy FFPE kit (Qiagen, Hilden, Germany). cDNA was synthesised from 1 μg of RNA using the Superscript II First Strand cDNA synthesis kit (Thermo Fisher Scientific), according to the manufacturer’s instructions. Transcript levels were analysed using SYBR Green MasterMix (Thermo Fisher Scientific) on a StepOne Real-Time PCR system (Applied Biosystems, Thermo Fisher Scientific), under standard cycling conditions. All samples were assayed in triplicate. Primer sequences are shown in Table S2.

### 4.8. SOD and CAT Activity Assay

SOD activity was determined using the SOD assay kit (Sigma; 19160) according to the manufacturer’s instructions. CAT activity was determined using the catalase activity assay kit (Abcam; ab83464), according to the manufacturer’s instructions.

### 4.9. Zebrafish Husbandry

wt, AB strain (wt), and *rdh12^u533^* zebrafish were bred and maintained according to local UCL and U.K. Home Office regulations for the care and use of laboratory animals under the Animals Scientific Procedures Act at the UCL Bloomsbury campus zebrafish facility. Zebrafish were raised at 28.5 °C on a 14 h light/10 h dark cycle. UCL Animal Welfare and Ethical Review Body approved all procedures for experimental protocols, in addition to the U.K. Home Office (License no. PPL PC916FDE7). All approved standard protocols followed the guidelines of the ARVO Statement for the Use of Animals in Ophthalmic and Vision Research Ethics. Zebrafish were terminally anaesthetised in 0.2 mg/mL Tricaine (MS−222) for sample collection.

### 4.10. CRISPR-Cas9 Generation of rdh12 Fish

CRISPR guides were designed using Benchlings CRISPR Guide Design Software, targeting exon 1 of rdh12, and the following guide was used: 5′-TGGCGTTCGCGGCGGGTTTAGGG. Mutagenesis was carried out as previously described [33]. Mutations were analysed by Sanger sequencing using the forward primer 5′-GGAGGCTGCTGAACACATTC and reverse primer 5′-CGATTTCTGGAGCAGATCATGTC. Injected zebrafish were raised to adulthood (F0) and outcrossed with wild-type fish to produce the F1 generation. F1 finclip DNA was assessed for heterozygous mutation carriers. These fish were then outcrossed with wild-type fish to produce an F2 generation. Heterozygous fish carrying the same mutation from the F2 generation were then in-crossed to produce the F3 generation. Homozygous fish and their wt siblings were used for all further characterisations.

### 4.11. Immunohistochemistry

Cryosections were prepared from adult enucleated eyes or whole embryos and immunostained as previously described [36]. Primary antibodies used were 4D2 (1:200; Abcam), 1D4 (1:200; Abcam), and anti-blue opsin (1:200; gifted by Professor David Hyde, University of Notre Dame). Slides were incubated with primary antibodies overnight at 4 °C, washed, and then incubated with appropriate Alex Fluor secondary antibodies (Thermo Fisher Scientific) for 2 h at RT. Slides were imaged on Leica LSM 710 confocal microscope.

### 4.12. TUNEL Assay

TUNEL assay was performed on cryosections using the ApopTag Plus Fluorescein In Situ Apoptosis Detection Kit (Merck-Millipore, Burlington, MA, USA), according to the manufacturer’s instructions. Slides were imaged on a Leica LSM 710 confocal microscope.

### 4.13. Retinal Histology

Whole embryos were fixed in 4% PFA/PBS overnight at 4 °C, then embedded using JB-4 embedding kit (Polysciences Inc., Warrington, PA, USA). Sections were cut at a thickness of 7 µm. For adult fish, enucleated eyes embedded for electron microscopy, as described below, were sectioned at a thickness of 1 µm. Sections were stained with 1% toluidine blue and imaged on an a Axioplan 2 microscope (ZEISS Microscopy, Jena, Germany).

### 4.14. HPLC Analysis of atRAL Levels in Zebrafish

Retinoids were extracted according to the protocol by Costaridis et al. [39]. Approximately 250 embryos or adult enucleated eyes were collected and sonicated in 1 mL stabilising buffer (PBS, 0.5% ascorbic acid, 0.5% EDTA, 0.3% sodium sulphate, pH 7.3). Sample was extracted twice in 2 mL extraction solvent (8:1 ethyl acetate:methyl acetate plus 0.5% butylated hydroxytoluene), with vigorous shaking for 30 min. Extracts were combined and dried under nitrogen gas and redissolved in acetonitrile. HPLC analysis was performed using a Waters ACQUITY^®^ UPLC^®^. Elution was monitored at 360 nm for atRAL with a Waters ACQUITY 2996 PDA. The stationary phase was Waters ACQUITY UPLC BEH C18 1.7 μm, 2.1 × 50 mm column and the mobile phase consisted of acetonitrile/water 0.1% formic acid (87.5:12.5). The analysis lasted 8 min, with a flow rate of 0.25 mL/min and a 10 μL injection.

### 4.15. Transmission Electron Microscopy

Enucleated eyes from 12 mpf zebrafish were fixed and embedded as previously described [33]. Blocks were sectioned using a UC7 ultramicrotome (Leica Microsystems) and 70 nm sections were picked up on Formvar-coated 2 mm slot copper grids (Gilder Grids Ltd., Grantham, UK) and post-stained with Reynolds lead citrate for 5 min. Sections were viewed using a 120 kV Tecnai G2 Spirit transmission electron microscope (FEI Company, Eindhoven, The Netherlands) and images were captured using a OneView UltraScan^®^ 4000 camera (Gatan Inc., Pleasanton, CA, USA) and Serial EM software [40]. The images were stitched together using IMOD [41]. Visualisation was done in 3dmod. Images were analysed using ImageJ.

### 4.16. Statistical Analysis

All statistical analysis was performed using GraphPad Prism 8. All data are expressed as mean ± SEM of at least three independent experiments. For all analyses, Shapiro–Wilk normality test was initially carried out to determine if data are normally distributed, and the appropriate statistical test was chosen. For comparison between two groups, data were analysed either using paired or unpaired *t*-test, depending on experimental design or Mann–Whitney test. For more than two groups, statistical significance was analysed using one-way ANOVA, followed by Sidak’s multiple comparison test. For grouped analyses, two-way ANOVA with Dunnett’s multiple comparison test was used. A *p*-value of <0.05 was considered significant.

## Data Availability

Data are contained within the article or Supplementary Materials.

## Supplementary Materials

The following are available online at https://www.mdpi.com/article/10.3390/ijms22168863/s1.
