# Supplementary material for: Involvement of Oxidative and Endoplasmic Reticulum Stress in RDH12-Related Retinopathies
Source: Int J Mol Sci. 2021 Aug 18;22(16):8863. doi: 10.3390/ijms22168863 (PMC8396253; doi:10.3390/ijms22168863)
Supplement: Supplementary file 1 [file ijms-22-08863-s001.zip › ijms-1327716-supplementary.pdf]

Supplementary material

Mutation	Protein change	Primers
**c.677A>G**	p.Y226C	5'-cggggtcaccacctgcgcagtgcac-3′<br>5'-gtgcactgcgcaggtggtgaccccg-3'
**c.325G>C**	p.A109P	5'-ctatccgagcctttcctgagggctttctgg-3'<br>5'-ccagaaagccctcaggaaaggctcggatag-3'
**c.38C>A**	p.S13*	5'-gctcacctccttcttctagttcctgtatatggtag-3'<br>5'-ctaccatatacaggaactagaagaaggaggtgagc-3'

Table S1: Primers used for site directed mutagenesis. The mutated base pair is shown in red.

Gene	Forward primer	Reverse primer	Reference
**Human**			
**ATF4**	TCAAACCTCATGGGTTCTCC	GTGTCATCCAACGTGGTCAG	
**CHOP**	GACCTGCAAGAGGTCCTGTC	TGTGACCTCTGCTGGTTCTG	
**sXBP1**	CTGAGTCCGAATCAGGTGCAG	ATCCATGGGGAGATGTTCTGG	
**GAPDH**	ACAGTTGCCATGTAGACC	TTTTTGGTTGAGCACAGG	
**NRF2**	CAACTACTCCCAGGTTGCCC	AAGTGACTGAAACGTAGCCGA	
**SOD2**	GCTGGAAGCCATCAAACGTG	GCAGTGGAATAAGGCCTGTTG	
**CAT**	CTCCGGAACAACAGCCTTCT	GAATGCCCGCACCTGAGTAA	
**GPX1**	CCGGGACTACACCCAGATGA	TCTTGGCGTTCTCCTGATGC	
**HO-1**	CTCCTCTCGAGCGTCCTCAG	AAATCCTGGGGCATGCTGTC	
**Zebrafish**			
**sod1**	GGGCCAACCGATAGTGTGAG	TCATCCTCCTTCTCATGAATCACC	
**sod2**	CAAGGGACCACAGGTCTCATC	TGGAAACGCTCGCTGACATT	
**cat**	AGGGCAACTGGGATCTTACA	TTTATGGGACCAGACCTTG	42
**gpx1**	TGAGGCACAACAGTCAGGGA	CATTCTTGCAGTTCTCCTGGTGC	
**hmox1a**	CACAGAGACTGAGAGAGATTGGC	TGCCCACTCCTAATGCGAAC	
**nrf2**	ATGTCTAAAATGCAGCCAAGCC	CGGTAGCTGAAGTCGAACAC	43
**atg5**	CCCTACTATCTGCTCCTCCCAC	GGAGGTCGAACAACACACCA	
**atg7**	AGAGTCCAGTCCGATGTC	GAAGTAACAGCCGAGCG	
**atg12**	CCAGTTCATCTCACGCTTCCTC	TGCCGTCACTTCCGAAACAC	
**p62**	TTTGGCTCTTGTGAAGGATGAC	GAGGGCTAAAGTGAGGTGTAGTGA	
**edem**	CGTTTCGGTGCTCTTCCTGAG	GCGTAACCACACCTCACTTTG	44
**bip**	CAAGAAGAAGACGGGCAAAG	CTCCTCAAACTTGGCTCTGG	44
**atf4**	TGAGCACACTGAGGTTCCAG	GTCTTCACTCGGCCTTTGAG	44
**atf6**	TGATGAGGCACTGTCTCCAG	ATGGGTCTTTTTGCTGGTTG	44
**chop**	ATATACTGGGCTCCGACACG	GATGAGGTGTTCTCCGTGGT	44
**xbp1-s**	TGTTGCGAGACAAGACGA	CCTGCACCTGCTGCGGACT	45
**actin**	CGAGCTGTCTTCCCATCCA	TCACCAACGTAGCTGTCTTTCTG	

Table S2: RT-qPCR primer sequences.

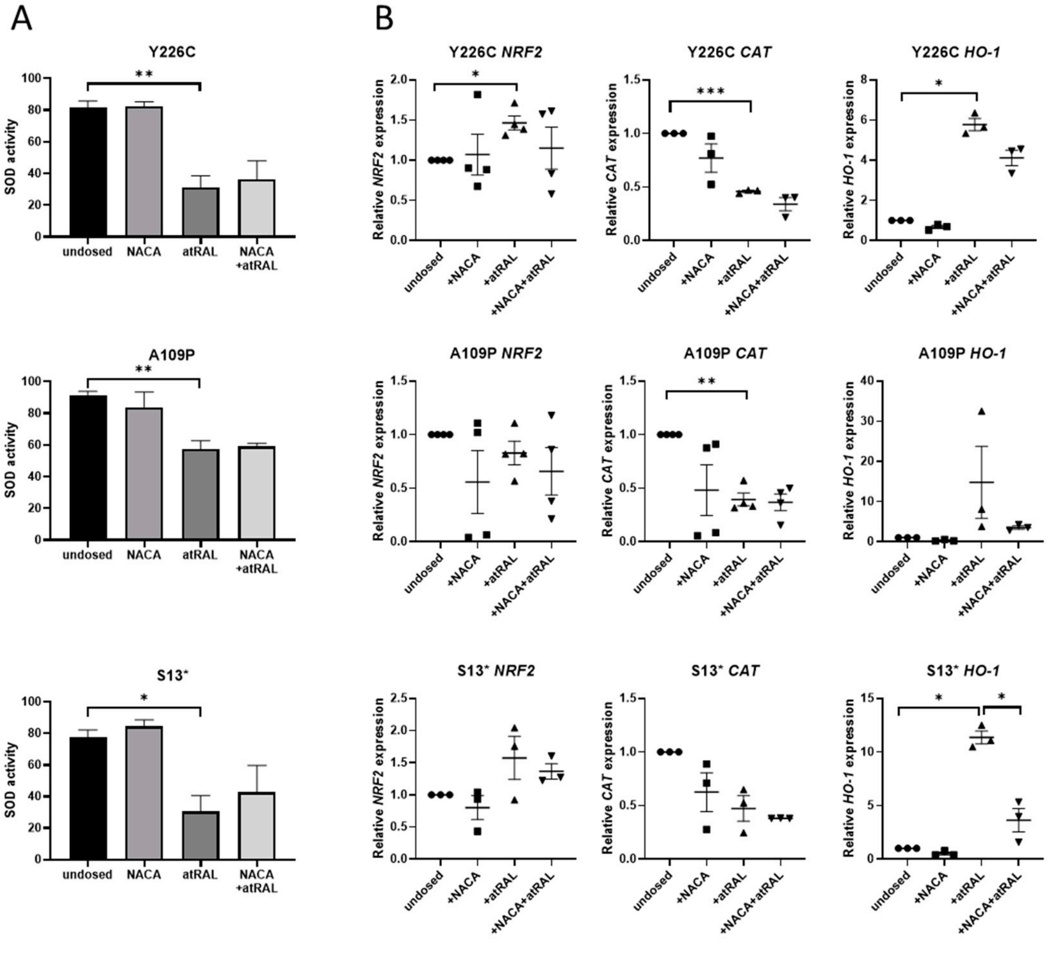

Figure S1: NACA does not attenuate atRAL induced oxidative stress. Cells were dosed with 50 μM atRAL, 750 μM NACA or both for 24 hours, then analysed by **(A)** SOD activity assay and **(B)** RT-qPCR of oxidative stress markers *NRF2*, *CAT* and *HO-1*. NACA reduced atRAL induced expression of HO-1 in the p.S13* line. Statistical significance was analysed using one-way ANOVA and Sidak's multiple comparison test. *p ≤0.05, **p ≤ 0.01, ***p ≤ 0.001.

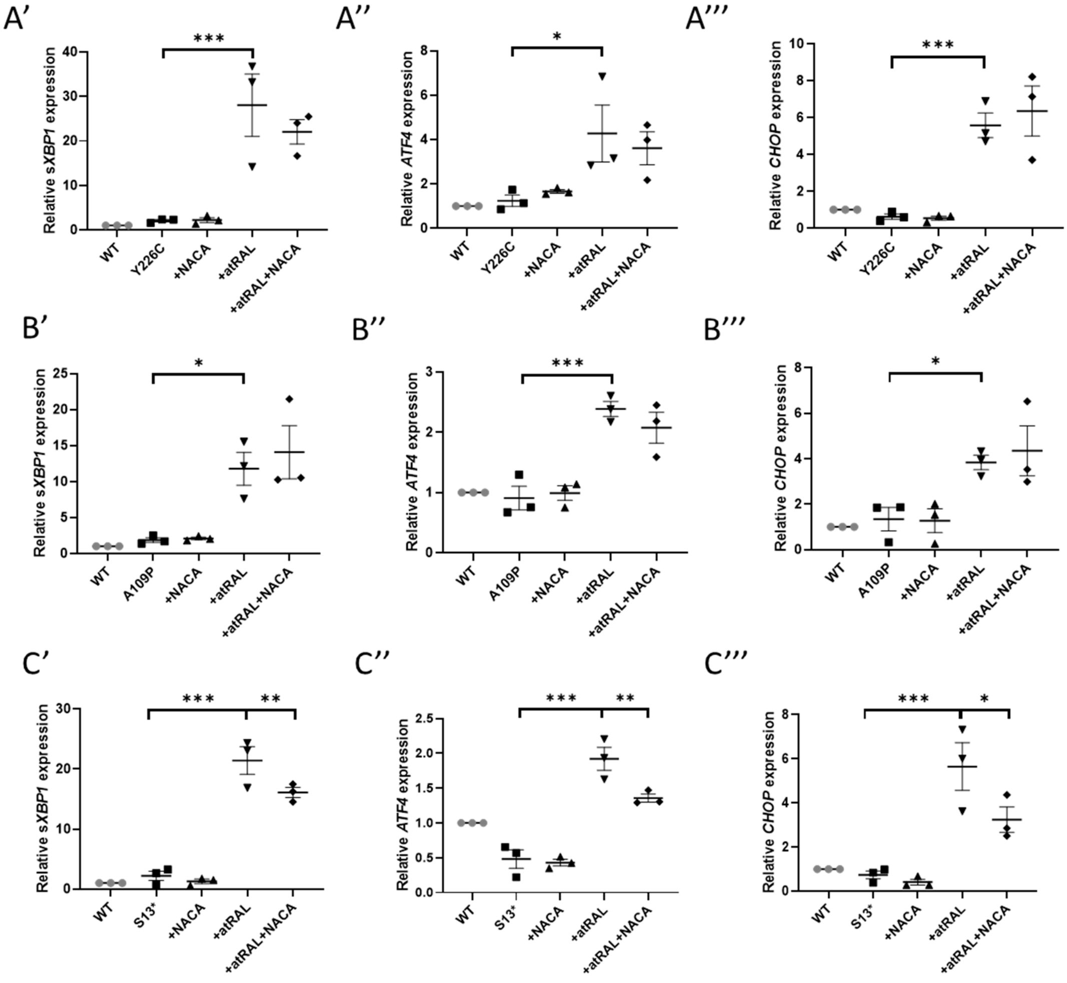

Figure S2: NACA reduces atRAL induced ER stress in the p.S13* cell line. Cells were dosed with 50 μM atRAL, 750 μM NACA or both for 24 hours. RT-qPCR was performed analysing the expression of ER stress markers. NACA did not significantly reduce expression of ER stress markers in p.Y226C or p.A109P cell lines. NACA significantly reduces expression of *XBP1*, *CHOP* and *ATF4* in p.S13* cell line. Statistical significance was analysed using one-way ANOVA and Sidak’s multiple comparison test. *$p \leq 0.05$, **$p \leq 0.01$, ***$p \leq 0.001$.

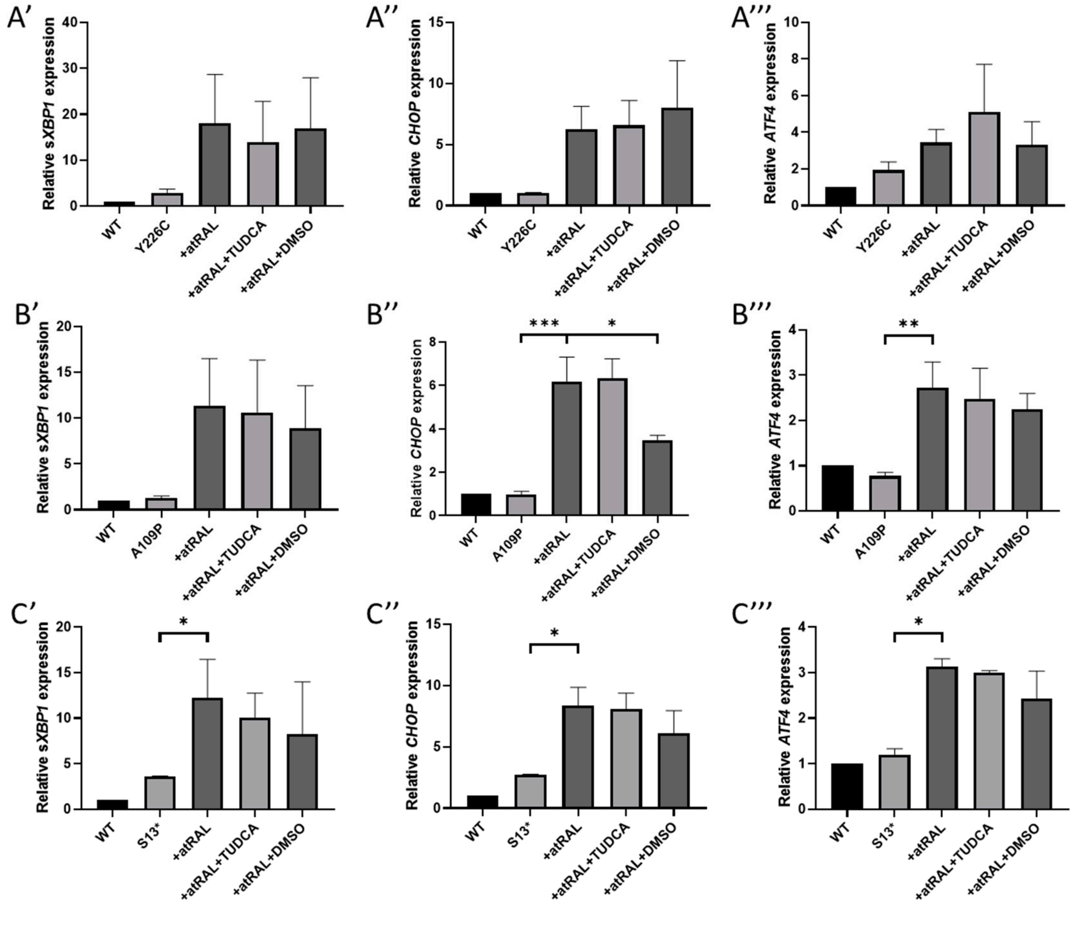

Figure S3: TUDCA does not reduce atRAL induced ER stress. TUDCA was prepared at a stock concentration of 20 mM in DMSO, and further diluted in culture media. Cells were dosed with 50 μM atRAL alone or together with 100 μM TUDCA or 0.5% DMSO for 24 hours. Expression of ER stress markers was analysed by RT-qPCR. Data are expressed as mean ± SEM. Statistical significance was analysed using one-way ANOVA and Sidak's multiple comparison test. *p ≤0.05, **p ≤ 0.01, ***p ≤ 0.001.

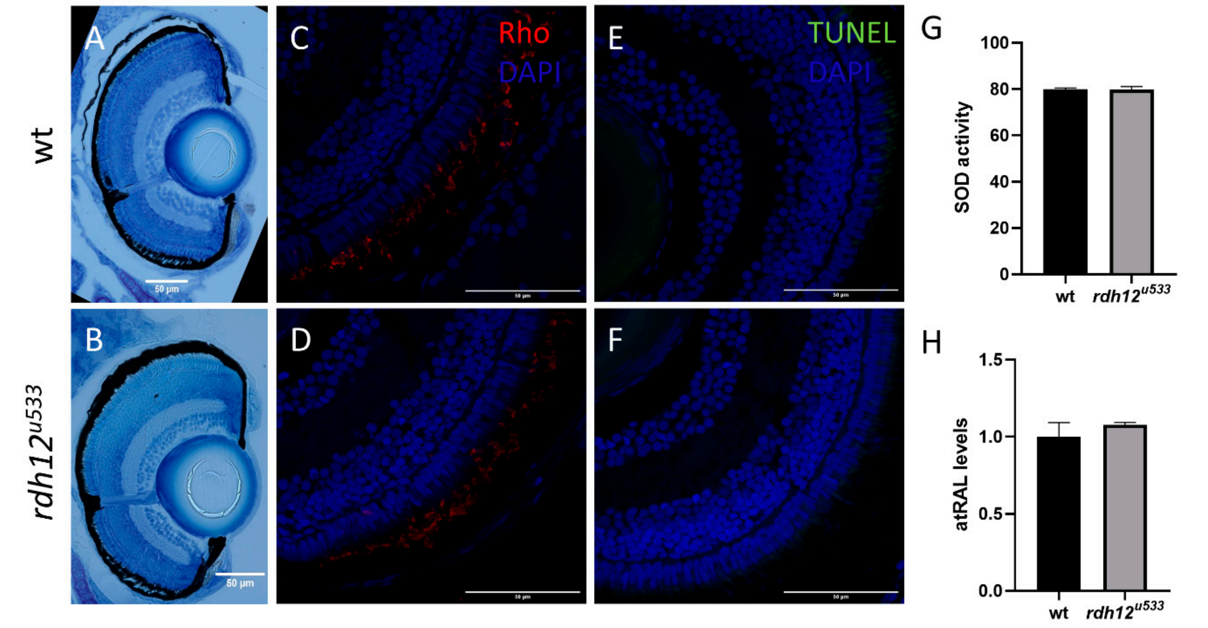

Figure S4: Characterisation of *rdh12^{u533}* zebrafish at 5 dpf. Retinal sections were stained with toluidine blue to assess retinal structure (A, B). Immunohistochemistry staining was used to detect rhodopsin (C, D). TUNEL assay was performed to detect cell death (E, F). Scale bar = 50 μM. (G) SOD activity was measured in wt and rdh12 fish at 5 dpf. (H) atRAL levels in wt and *rdh12^{u533}* fish were analysed using HPLC. No differences between wt and *rdh12^{u533}* fish were detected at 5 dpf.

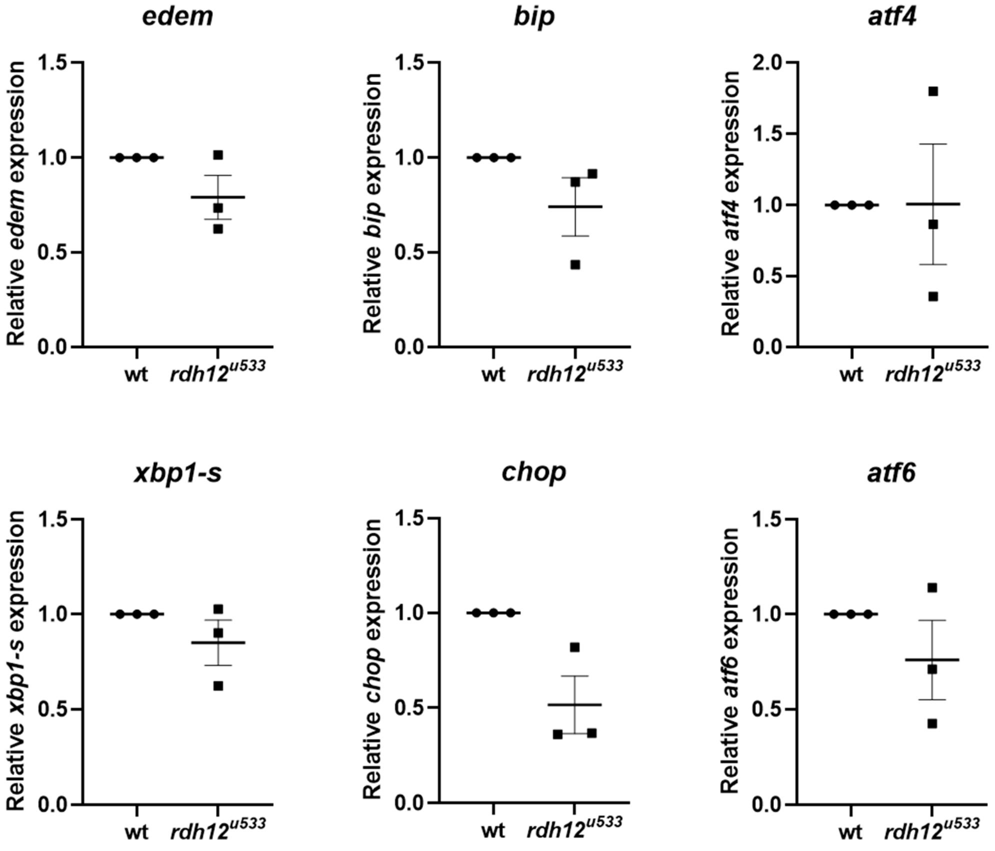

Figure S5: ER stress is not disrupted in *rdh12*^{u533} mutant fish. Expression of ER stress markers were analysed by RT-qPCR in 12 mpf retinas. No significant differences in expression between wt and *rdh12*^{u533} fish was seen. Data are expressed as mean ± SEM.

References

42. Wang, X.; Wei, L.; Wang, Y.; He, B.; Kong, B.; Zhu, J.; Jin, Y.; Fu, Z. Evaluation of development, locomotor behavior, oxidative stress, immune responses and apoptosis in developing zebrafish (Danio rerio) exposed to TBECH (tetrabromoethylcyclohexane). *Comp. Biochem. Physiol. Part C Toxicol. Pharmacol.* **2019**, *217*, 106–113, doi:10.1016/j.cbpc.2018.12.004.
43. Nguyen, V.T.; Fuse, Y.; Tamaoki, J.; Akiyama, S.-I.; Muratani, M.; Tamaru, Y.; Kobayashi, M. Conservation of the Nrf2-Mediated Gene Regulation of Proteasome Subunits and Glucose Metabolism in Zebrafish. *Oxidative Med. Cell. Longev.* **2016**, *2016*, 5720574, doi:10.1155/2016/5720574.
44. Park, J.-S.; Song, J.; Park, J.-S.; Lee, S.; Lee, J.; Park, H.-J.; Kim, W.-K.; Yoon, S.; Chun, H.-S. 3,4-Dichloroaniline promotes fatty liver in zebrafish larvae. *Mol. Cell. Toxicol.* **2020**, *16*, 159–165, doi:10.1007/s13273-019-00066-5.
45. Vacaru, A.M.; Di Narzo, A.F.; Howarth, D.L.; Tsedensodnom, O.; Imrie, D.; Cinaroglu, A.; Amin, S.; Hao, K.; Sadler, K.C. Molecularly defined unfolded protein response subclasses have distinct correlations with fatty liver disease in zebrafish. *Dis. Models Mech.* **2014**, *7*, 823–835, doi:10.1242/dmm.014472.
